# Blue Zones-Based Worksite Nutrition Intervention: Positive Impact on Employee Wellbeing

**DOI:** 10.3389/fnut.2022.795387

**Published:** 2022-02-11

**Authors:** Ciara Heath, Nanette V. Lopez, Valerie Seeton, Jay T. Sutliffe

**Affiliations:** ^1^Department of Health Sciences, Northern Arizona University, Flagstaff, AZ, United States; ^2^Plant Rich and Nutrient Dense Interventions for Active Lifestyles (PRANDIAL) Laboratory, Department of Health Sciences, Northern Arizona University, Flagstaff, AZ, United States; ^3^Family Consumer and Health Sciences, Coconino Cooperative Extension, Arizona Health Zone, University of Arizona, Flagstaff, AZ, United States

**Keywords:** plant-based, mental wellness, dietary quality, blue zones, virtual intervention

## Abstract

“Blue Zones” are geographical regions where people live to be non-agenarians and centenarians with significantly better rates of mental wellness when compared to the average American. It was discovered that these areas have nine unique evidenced-based lifestyle principles, with one of their main principles being the consumption of a plant-based diet. With this in mind, we performed a worksite intervention with the objective of understanding the relationships among Blue Zones knowledge, a plant-based lifestyle, and improvements in overall mental wellness during the COVID-19 pandemic. During spring 2021, we recruited 52 employees from a public, mid-sized university in the southwestern United States to participate in an 8-week virtual intervention that included weekly topic presentations, cooking demonstrations, and Blue Zones education. Participants were also assigned to weekly wellness counseling groups integrating Motivational Interviewing based principles that included additional, relevant conversation topics and support. The final sample (*n* = 52 participants) had a mean age of 45.6 ± 10.6 years. Participants were predominantly women (84.6%) and nearly half were married (44.2%). The majority attended graduate school (59.6%) and identified as White (84.6%). Paired-samples *t*-tests indicated significant improvements in all mental wellness outcomes and Healthy Eating Index-2015 (HEI-2015) scores over time (*p's* < 0.001 to 0.02). Multiple linear regression models revealed that Blue Zones knowledge (β = −0.037, *p* = 0.010) significantly negatively predicted Patient Health Questionnaire-9 (PHQ-9) scores at 8-weeks. Additionally, multiple linear regression models indicated small group attendance (β = −1.51, *p* = 0.003) and Blue Zones knowledge (β = −0.81, *p* = 0.012) significantly negatively predicted sleep scores at 8-weeks. When HEI-2015 total scores were also included at baseline and 8-weeks (post-intervention), Blue Zones knowledge (β = −0.031, *p* = 0.049) was a borderline significant predictor of PHQ-9 at 8-weeks. Additionally, small group sessions (β = −1.52 *p* = 0.005) were a significant predictor of sleep at 8-weeks. The intervention illustrated that virtual intervention strategies can improve nutrition and mental wellness for future advancement in life quality and wellbeing.

## Introduction

Mental health conditions in the U.S. are a major public health concern. Nearly one in five U.S. adults live with a mental health condition, which amounts to 51.5 million people (1). Depression, a frequently diagnosed mental health condition, has significantly increased from 2005 to 2015 (2). Concomitant with the rise in depression, the economic burden of major depressive conditions has increased 21.5% over the same period (3). Those suffering from mental health conditions struggle with activities of daily living, and have negative alterations in their mood and behaviors. A mental health condition can present as emotional and physical symptoms such as depressed mood, loss of interest in things previously enjoyed, and reduced energy leading to decreased activity (4). Treatment for mental health conditions remains infrequent and is often inadequate (5). As a result, mental health conditions are the leading cause of disability and mortality worldwide (4), leading to suicide, which is the tenth leading cause of death among US adults (6).

In addition to the stressors already existing in our society, the COVID-19 pandemic has exacerbated living conditions. Impacts on health, infringement on freedom, significant financial losses, unfamiliar public health measures, and conflicting messages from authorities have caused increased emotional distress and increased risk for mental illness (7). Working and leisure lifestyles switched to virtual formats, with many travel restrictions imposed. Increased rates of anxiety, depression, post-traumatic stress disorder, psychological distress, and stress have been reported (8), worsening existing mental health problems.

Solutions to these mental health burdens may include therapeutic lifestyle factors such as exercise, proper nutrition, time in nature, supportive relationships, recreation, relaxation, and spiritual involvement (9). “Blue Zones” include five areas where people follow a unique set of nine evidence-based lifestyle principles, living significantly longer than the rest of the world, with significantly better mental health and quality of life (10, 11). These nine principles include: move naturally, live with purpose, slow down in life, stop eating once you are 80% full, drink 1–2 glasses of wine a day, find a sense of belonging, put your loved ones first, find your social circle, and one of their main principles, consume a plant-based diet. These principles contribute substantially to individuals' improved mental health, overall wellness, and longevity (12). Because of these positive health outcomes, researchers are turning to the Blue Zone principles for answers on improving overall mental wellness, explicitly focusing on their diets (13).

Individuals with greater depressive symptoms often have lower diet quality, indicating that diet may play a major role in mental health (14). Research evaluating the Blue Zones and their principles illustrate the benefits of a plant-based diet on mental health and wellbeing. Previous research indicates that wellbeing increases with overall consumption of fruits and vegetables and with increasing the number of days fruits and vegetables are consumed (15, 16). Results from interventions encouraging individuals to increase consumption of plant-based food options also found positive improvements on overall mental health and wellbeing, reducing depressive symptoms and improving sleep quality (17, 18). A causal relationship between a plant-based diet and positive mental health outcomes is less established, especially when compared to the extant literature that supports the positive effects of a plant-based intervention on physical health (19). Further research is needed to assess the health outcomes of worksite nutrition interventions (20).

Using the nine Blue Zones principles as a basis for our pilot study, we examined the relationship between a plant-based diet and mental health and wellbeing, while incorporating the virtual lifestyle program during the ongoing COVID-19 pandemic. The objective was to administer a virtual worksite intervention to support healthy lifestyle behavior changes, including implementing a plant-based diet, as worksite interventions are found to increase work productivity and demonstrate improvements in health behaviors such as increasing physical activity and improveming in diet (20, 21). We assessed the following aims: (1) if this online intervention is effective at improving diet and mental wellness (2) if attendance at weekly workshops and small group sessions and Blue Zones Knowledge impacted changes in mental wellness over time, and (3) if the addition of diet quality elicited differences overtime for all mental wellness outcomes.

## Materials and Methods

### Study Design

We conducted an 8-week virtual worksite nutrition intervention using a pre-post experimental design. All participants attended weekly evening sessions consisting of an educational presentation and a cooking demonstration, which could be watched in real time or via a recording. Participants were given all recipes in advance and encouraged to purchase the ingredients to cook the meals simultaneously as the class. In addition, all participants received weekly emails that included plant-based recipes, Blue Zones principles information, and an optional goal-setting activity. Participants were assigned to a group that included weekly wellness counseling group sessions guided by an individual trained in motivational interviewing.

### Study Setting

Participants completed intervention components virtually, using the Zoom platform. The weekly live-streamed evening session occurred in a privately owned residence kitchen and was recorded for participants who could not attend. The recorded sessions were uploaded to an external website that tracked attendance and prompted questions as participants were viewing to verify people were paying attention. The weekly wellness counseling group sessions were held virtually via Zoom. Baseline and post-intervention surveys were distributed via Qualtrics, an online platform.

### Study Population and Recruitment

Recruited participants were (blinded for review - midsize university) employees, aged 18 years or older, and were interested in learning more about applying plant-based principles into their lifestyle. Participants were excluded if they were currently consuming an exclusively plant-based diet or participating in a weight loss program, determined through a pre-intervention questionnaire. Participants agreed to attend either the weekly evening session live through Zoom or be willing to watch the recorded session at a later time.

Participants were recruited through the Employee Assistance and Wellness (EAW) program, an employee support program at the university that focuses on improving and ensuring the health and wellness of their employees (22). They received two email blast invitations for participation in the study. To recruit more participants, an additional email blast was distributed through listservs to various staff networks, including service professionals on campus. Word of mouth was also used to recruit additional study participants. Participants were followed for 8-weeks, with further follow-up planned next year (Spring 2022). Due to the exploratory nature of the pilot study, power analyses to determine required sample size were not conducted. Instead, one of the study goals included the feasibility of conducting a virtual nutrition intervention.

As an incentive to participate in the study, participants received up to 200 HealthyU points through the EAW program, which they can cash out for a cash reward in February of 2022. They were also given a copy of a plant-based cookbook, and entered into a raffle at the end to win one of three gift cards to a local grocery store upon completion of all intervention tasks, including attending all sessions live or recorded.

### Assessments

Mental wellness outcomes were assessed via changes from baseline to post-intervention using multiple measures. Surveys assessed depression (PHQ-9), general anxiety disorder (GAD-7), sleep patterns, social relationships, satisfaction with life (SWLS), and perceived stress (PSS-10). The Patient Health Questionnaire (PHQ-9) is a 9-item validated questionnaire assessing the frequency of depression symptoms on a scale of 0 (not at all) to 3 (nearly every day), with total scores ranging from 0 to 27 (α = 0.787) (23). Higher scores indicate more depression symptoms. The GAD-7 is a 7-item validated questionnaire assessing the frequency of anxiety symptoms on a scale of 0 (not at all) to 3 (nearly every day) with total scores ranging from 0 to 27 (α = 0.855) (24). Higher scores indicate more anxiety symptoms. The sleep assessment is a 15-item questionnaire assessing frequency of sleep patterns on a scale of 1 (never) to 5 (always) and 4 yes or no questions regarding sleep quantity, night shift work, and medical conditions affecting sleep, with total scores ranging from 11 to 59. Lower scores indicate more healthful sleep patterns. The social relationships assessment is an 8-item questionnaire assessing the frequency of relationship purpose and meaning on a scale ranging from 1 (never) to 5 (always) with a total score ranging from 8 to 40 (α = 0.848). Higher scores indicate more purposeful and meaningful social relationships. The Satisfaction with Life (SWLS) is a 5-item validated questionnaire assessing subjective wellbeing on a scale of 1 (strongly disagree) to 7 (strongly agree) with final scores ranging from 5 to 35 (α = 0.880) (25). Higher scores indicate greater satisfaction with life. The Perceived Stress Scale (PSS-10) is a 10-item questionnaire assessing the degree of stress experienced in everyday life during the past month on a scale of 0 (never) to 4 (very often) with total scores ranging from 0 to 40 (α = 0.872). Higher scores indicate greater perceived stress. The sleep assessment, social relationships assessment, and perceived stress survey were all obtained from an American College of Lifestyle Medicine survey (26).

Diet quality outcomes were assessed via changes from baseline to post-intervention. Mean Healthy Eating Index-2015 (HEI-2015) scores were determined upon completion of three online, self-administered 24-h dietary recalls (ASA-24) conducted at each time point. The ASA-24 is a standardized and validated dietary assessment tool that guides participants through a 24-h dietary recall, asking detailed questions to help limit missingness (27). HEI-2015 scores range on a scale from 0 to 100, with higher scores equating to overall better diet quality based on the Dietary Guidelines for Americans. Three 24-h diet recalls were collected in order to capture an accurate estimate of an individual's usual dietary intake (28, 29). In addition, participants were strongly encouraged to complete 2 weekdays and 1 weekend diet recall in order to fully understand their dietary choices. Blue Zones knowledge was assessed post-intervention within the mental health and wellness survey. The question stated, “How knowledgeable do you feel about the Blue Zones principles?” Responses were self-reported as a continuous variable on a scale of 0 to 100.

Sociodemographic information was collected at baseline and included: age, gender, race, income, employment status, education, and marital status. Age and income were collected as a continuous variable, sex as a dichotomous variable, and race, income, employment status, education, and marital status were collected as categorical data. Other data collected included participants' work departments, physical activity level, smoking status, and prescription medications used. Adherence to the intervention was determined through attendance at the weekly evening Zoom sessions, as well as attendance to the wellness counseling group sessions (group A). A member of the research team was responsible for tracking attendance at every session.

### Intervention Educational Topics/Materials

Six educational sessions detailing a plant-based lifestyle were given weekly throughout the intervention and covered various topics including Blue Zones, mental health, physical activity, and the environment. The educational sessions started on the second week and occurred weekly thereafter, ending on the seventh night. The educational sessions were modeled using previously published research topics and lasted 45 min. The first through sixth session topics included: how to incorporate more plant-based foods into one's diet, the effects of consuming plant-based foods on the environment, general overview of Blue Zones, incorporating spices into food preparation, the role of diet on mental health, and the impact of diet on fitness. For the first and final sessions, Joel Fuhrman, MD, a lifestyle medicine physician and expert on plant-based lifestyles, led question and answer sessions. All sessions were moderated by a Registered Dietitian Nutritionist (RDN), which has previously positively impacted other worksite wellness programs (20). Six one-page handouts detailing the Blue Zones Principles were provided to the participants via email throughout the study. Each handout focused on a single principle and included information supported by previous research regarding the topic.

Motivational Interviewing (MI) was utilized in the counseling group sessions to assist individuals in adopting a plant-based lifestyle. MI has become a popular, effective counseling method to help produce effective, attainable health behavior changes, including increased consumption of fruits and vegetables (30). This study added a MI-based weekly wellness counseling group session to help individuals implement plant-based lifestyle habits into their routine.

### Statistical Analyses

Means and standard deviations were calculated for all continuous variables. Frequencies were determined for all categorical variables. Paired samples *t*-tests were conducted to determine if knowledge of Blue Zones principles and a plant-based lifestyle intervention paired with weekly wellness coaching elicited differences overtime for all mental wellness outcomes and dietary quality. To determine if attendance at weekly workshops and small group sessions impacted changes in mental wellness over time, multiple linear regression models were used to examine mental wellness scores at 8-weeks, using baseline mental wellness scores as covariates, and small group session attendance and workshop attendance as predictors. To examine if a plant-based lifestyle intervention impacts mental health among employees, multiple linear regression models were used to examine mental wellness scores at 8-weeks, using baseline mental wellness scores and HEI-2015 scores at baseline as predictors and 8-weeks mental wellness scores as covariates. All analyses were conducted using SPSS v27.

## Results

Sociodemographic characteristics of participants (*n* = 52) are presented in Table 1. Participants were primarily female (84.6%) and White (84.6%) and completed graduate studies (59.6%). Paired samples *t*-tests evaluating differences between baseline and 8-weeks (post-intervention) for all mental wellness outcomes (i.e., PHQ-9, GAD, Sleep, Social Relationships, SWLS, PSS-10) were statistically significant (range *p's* < 0.001 to 0.02) (Table 2). The paired samples *t*-test evaluating differences in diet quality between baseline and 8-weeks (post-intervention) was statistically significant, with greater than a 12-point improvement in HEI-2015 total scores at 8-weeks (*p* < 0.001) (Table 2).

**Table 1:** Sociodemographic characteristics of participants at baseline (*n* = 52).

**Socio-demographics**	**Mean (std. dev.)**
Age (years)	45.62 (10.58)
**Sex**	*n* (%)
Male	8 (15.4)
Female	44 (84.6)
**Marital Status**	
Married	23 (44.2)
Single or non-partnered	29 (55.8)
**Education**	
Some college or college graduate	21 (40.4)
Completed graduate or professional school	31 (59.6)
**Ethnicity**	
Hispanic	6 (11.5)
Not hispanic	46 (88.5)
**Race**	
Caucasian/white	44 (84.6)
African-American/black	1 (1.9)
Asian-American/Asian	1(1.9)
Native American/Alaskan Native	2 (3.8)
Multi-racial	3 (5.8)
Unknown	1 (1.9)
**Income**	
$25,000–44,999	8 (15.4)
$45,000–74,999	20 (38.5)
$75,000–94,999	8 (15.4)
$95,000–124,999	9 (17.2)
$125,000+	7 (13.5)

**Table 2:** Mean values of mental wellness and diet quality scores at baseline and 8-weeks (post-intervention).

**Variable**	**Baseline mean** **(SD)**	**8-weeks** **mean (SD)**	***t***	***p*-value**
PHQ-9 (*n* = 43)	3.74 (2.78)	2.63 (2.04)	3.30	0.002
GAD (*n* = 52)	11.22 (3.47)	9.37 (3.22)	4.50	<0.001
Sleep (*n* = 50)	26.12 (4.79)	23.84 (4.86)	3.07	0.004
Social relationships (*n* = 52)	32.76 (5.13)	33.86 (4.85)	−2.60	0.012
SWLS (*n* = 50)	25.44 (5.02)	26.98 (6.00)	−2.38	0.021
PSS-10 (*n* = 50)	10.46 (4.32)	8.30 (4.13)	3.85	<0.001
HEI-2015 Total Score (*n* = 47)	62.43 (12.45)	75.15 (10.30)	−8.71	<0.001

Continuing to Table 3, results from regression analyses are presented. Multiple linear regressions examining mental wellness scores at 8-weeks, using baseline mental wellness scores as covariates, and small group session attendance, workshop attendance, and Blue Zones knowledge as predictors, indicated Blue Zones knowledge (β = −0.037, *p* = 0.010) significantly negatively predicted PHQ-9 scores at 8-weeks. Additionally, small group attendance (β = −1.51, *p* = 0.003) and Blue Zones knowledge (β = −0.81, *p* = 0.012) significantly negatively predicted sleep scores at 8-weeks. Small group session attendance, workshop attendance, and Blue Zones knowledge were not significant predictors of other mental wellness outcomes at 8-weeks (i.e., GAD, Social Relationships, SWLS, PSS-10). When linear regression models included HEI-2015 total scores at baseline and 8-weeks (post-intervention), Blue Zones knowledge (β = −0.031, *p* = 0.049) was a borderline significant predictor of PHQ-9 at 8-weeks. Additionally, small group sessions (β = −1.52 *p* = 0.005) were a significant predictor of sleep at 8-weeks. Small group session attendance, workshop attendance, and Blue Zones knowledge were not significant predictors of other mental wellness outcomes at 8-weeks.

**Table 3:** Linear regression associations between attendance at workshops, attendance in small group sessions, Blue Zones knowledge, HEI-2015 scores and baseline mental wellness scores with mental wellness outcomes at 8-weeks post-intervention among all participants in Blue Zones-based worksite intervention (*n* = 52).

**8-week Outcomes**							
			***R* ^2^**	**B**	**SE**	**95% CI**	***p*** **-** **value**
PHQ-9	PHQ-9 (BL)		0.462	0.449	0.088	0.270, 0.628	<0.001
	WS Attend			0.043	0.175	−0.311, 0.397	0.808
	SG Attend			−0.187	0.199	−0.591, 0.217	0.355
	BZ Knowl			−0.037	0.014	−0.065, −0.010	0.010
		PHQ-9 (BL)	0.518	0.391	0.100	0.186, 0.595	<0.001
		WS Attend		0.139	0.188	−0.243, 0.522	0.464
		SG Attend		−0.271	0.209	−0.695, 0.154	0.204
		BZ Knowl		−0.031	0.015	−0.063, 0.000	0.049
		HEI-2015 (BL)		−0.035	0.025	−0.087, 0.016	0.170
		HEI-2015 (8 wk)		0.000	0.032	−0.066, 0.066	0.998
GAD	GAD (BL)		0.283	0.446	0.113	0.218, 0.673	<0.001
	WS Attend			−0.199	0.251	−0.704, 0.306	0.431
	SG Attend			0.276	0.301	−0.331, 0.883	0.364
	BZ Knowl			−0.016	0.020	−0.055, 0.024	0.424
		GAD (BL)	0.380	0.418	0.138	0.139, 0.697	0.004
		WS Attend		−0.228	0.258	−0.750, 0.293	0.381
		SG Attend		0.137	0.300	−0.470, 0.745	0.649
		BZ Knowl		−0.013	0.021	−0.056, 0.030	0.535
		HEI-2015 (BL)		−0.053	0.038	−0.130, 0.023	0.166
		HEI-2015 (8 wk)		−0.043	0.048	−0.140, 0.055	0.382
Sleep	Sleep (BL)		0.416	0.451	0.118	0.213, 0.689	<0.001
	WS Attend			−0.257	0.397	−10.06, 0.542	0.520
	SG Attend			−1.51	0.477	−2.47, −0.551	0.003
	BZ Knowl			−0.081	0.031	−0.144, −0.019	0.012
		Sleep (BL)	0.381	0.441	0.138	0.162, 0.719	0.003
		WS Attend		−0.038	0.444	−0.937, 0.862	0.933
		SG Attend		−1.52	0.509	−2.55, −0.485	0.005
		BZ Knowl		−0.059	0.036	−0.131, 0.013	0.106
		HEI-2015 (BL)		−0.027	0.064	−0.157, 0.102	0.671
		HEI-2015 (8 wk)		−0.066	0.083	−0.234, 0.101	0.428
Social Relationships	Soc Rel (BL)		0.695	0.726	0.092	0.542, 0.911	<0.001
	WS Attend			−0.038	0.299	−0.640, 0.565	0.900
	SG Attend			−0.041	0.353	−0.752, 0.669	0.907
	BZ Knowl			0.042	0.023	−0.005, 0.088	0.080
		Soc Rel (BL)	0.696	0.756	0.106	0.542, 0.970	<0.001
		WS Attend		0.017	0.327	−0.645, 0.680	0.958
		SG Attend		0.34	0.374	−0.724, 0.792	0.927
		BZ Knowl		0.036	0.028	−0.021, 0.093	0.210
		HEI-2015 (BL)		−0.027	0.048	−0.123, 0.070	0.578
		HEI-2015 (8 wk)		0.069	0.062	−0.057, 0.195	0.274
SWLS	SWLS (BL)		0.462	0.786	0.136	0.513, 1.06	<0.001
	WS Attend			−0.354	0.480	−1.32, 0.613	0.464
	SG Attend			−0.020	0.584	−1.20, 1.16	0.973
	BZ Knowl			0.011	0.038	−0.064, 0.087	0.761
		SWLS (BL)	0.467	0.768	0.155	0.453, 10.08	<0.001
		WS Attend		−0.507	0.528	−1.58, 0.563	0.343
			***R* ^2^**	**B**	**SE**	**95% CI**	***p*** **-** **value**
		SG Attend		0.064	0.633	−1.22, 1.35	0.920
		BZ Knowl		−0.008	0.044	−0.097, 0.080	0.853
		HEI-2015 (BL)		0.053	0.080	−0.109, 0.214	0.512
		HEI-2015 (8 wk)		0.074	0.102	−0.132, 0.280	0.473
PSS-10	PSS-10 (BL)		0.334	0.526	0.118	0.289, 0.763	<0.001
	WS Attend			0.242	0.361	−0.486, 0.970	0.507
	SG Attend			−0.327	0.429	−1.19, 0.536	0.449
	BZ Knowl			−0.035	0.028	−0.092, 0.021	0.214
		PSS-10 (BL)	0.407	0.501	0.129	0.240, 0.763	<0.001
		WS Attend		0.241	0.379	−0.527, 1.01	0.529
		SG Attend		−0.467	0.434	−1.35, 0.413	0.289
		BZ Knowl		−0.027	0.031	−0.089, 0.035	0.384
		HEI-2015 (BL)		−0.031	0.056	−0.144, 0.082	0.582
		HEI-2015 (8 wk)		−0.096	0.070	−0.239, 0.046	0.178

*BL, baseline; WS, Workshop; SG, Small Group; BZ Knowl, Blue Zones Knowledge; HEI-2015, Health Eating Index-2015 Total Scores; PHQ-9, Physical Health Questionnaire; GAD, Generalized Anxiety Disorder; SWLS, Satisfaction with Life Scale; PSS-10, Perceived Stress Scale*.

## Discussion

With the benefits of consumption of increased plant-based foods and improved overall mental wellness becoming more established, and the shift toward virtual lifestyles during COVID-19 becoming more prevalent, the present study aimed to determine if a virtual worksite intervention would help individuals increase consumption of plant-based foods, adopt healthy lifestyle changes and see improvements in their overall mental wellness. In an attempt to make these changes, we provided education and support in a virtual format to help individuals be successful. As a result, individuals may be more successful and productive at work, as established in previous research (21, 31).

### Overall Intervention Effects

There were significant improvements in mental wellness over the 8 weeks, as seen through all mental health measures, showing that virtual interventions addressing dietary behavior change can be effective at improving mental wellness. Results also showed significant improvements in HEI-2015 scores, showing that diet quality improved over 8 weeks. These results are similar to other interventions of this nature (18). These improvements support the first aim, demonstrating that virtual dietary interventions during COVID-19 can improve mental wellness and diet quality.

Positive improvements in mental wellness could have been attributed to numerous reasons. By encouraging the participants in the study to eat a predominantly plant-based diet, potential improvements in their physical health could have positive impacts on mental health (32). For example, a recent study found that those with improved health behaviors are found to have improved mental health including stress, depression, and problems with emotions (33). In addition, there were multiple social aspects of the study that could have positively impacted the mental health outcomes. These include connectedness through the social online environment during weekly meetings, or the MI based small groups, which helped participants to set attainable goals for their lifestyle.

### Attendance, Blue Zones Knowledge, Diet Quality, and Mental Wellness

Attendance at weekly workshops and small group sessions paired with Blue Zones knowledge were analyzed as predictors of mental wellness outcomes. The results reveal that as small group attendance increased and Blue Zones knowledge improved, sleep quality scores improved. The increased research supporting the impact that sleep has on health (34) provides supporting evidence that the intervention helped to improve health overall, potentially providing a protective measure against COVID-19. While attendance and Blue Zones knowledge did not predict other mental wellness scores, attendance at virtual interventions should be strongly encouraged to ensure participants obtain all education and information possible, as well as have a positive impact on their sleep.

Diet quality was included as a predictor, to assess if there was a stronger improvement in mental wellness paired with attendance and Blue Zones knowledge. HEI-2015 scores did not predict mental wellness scores, and there were no significant relationships seen with any mental wellness scores. While there were no relationships, we still saw significant improvements in diet quality and mental wellness throughout the intervention, showing that the intervention itself was effective at improving both. In addition, when the HEI-2015 score was added into the models, we saw improvements in *R*^2^ for all mental wellness surveys besides sleep. This shows that the addition of HEI-2015 scores accounted for some of the variance seen with the mental wellness outcomes.

While attendance, Blue Zones Knowledge, and HEI-2015 did not significantly predict most mental wellness scores (anxiety, social relationships, satisfaction with life, and stress) there was likely another unmeasured variable that was a predictor of the positive changes we saw in the scores over time. The unmeasured variable could have been self-efficacy, family/friend support, or attitudes or intentions related to the skills/material. Additionally, we did not assess the frequency of use of the goal-setting activities that were encouraged but not mandatory for participants to complete. These variables should be studied in future interventions of this nature. Although we did not see positive improvements in Blue Zones Knowledge and mental wellness scores, participants reported positive feedback of following the Blue Zones principles. Thus, we are confident that these principles helped play a role in the improvements in their mental health, in conjunction with other aspects of the study as seen similarly in Blue Zones (12).

### Strengths and Limitations

There were multiple strengths of the study, but there are also weaknesses that must be noted. Due to the experimental study design, we were able to study the predictive effects of Blue Zones principles and implementing a plant-based lifestyle. Data were collected using validated and reliable surveys, providing acceptable results. Although the study was a pilot study and was completed to determine the effectiveness of a worksite intervention in a virtual environment, we attempted to recruit an adequate number of participants. Due to the virtual nature of this study, the total cost was reasonably inexpensive. In addition, the virtual format allows for widespread implementation and increases flexibility for participants as they can participate from different locations or with family members, allowing for increased support. This reinforces the increased use of online interventions for addressing dietary behavior change and improving mental wellness.

There were also sources of potential bias that need to be noted. Selection bias may be present due to participants volunteering to be part of the study. To reduce the selection bias, there were broad inclusion criteria and limited exclusion criteria to recruit a range of socio-demographically diverse individuals. Prior to enrollment 55% of participants indicated that they were moderately confident or extremely confident with incorporating a plant-based lifestyle into their daily routine, potentially contributing to the positive results that were seen. Approximately 85% of participants were female, as seen with previous studies using similar intervention designs. A predominantly female population tends to participate more in interventions. Finally, while we used primarily reliable and validated surveys (ASA-24 and mental wellness surveys) (23–25, 27), and the ASA-24 prompts participants to input additional data, there could be recall bias, resulting in systematic error due to differences in accuracy or completeness of past recalls. In addition, self-reporting of mental health may result in bias because, if someone was feeling “good” on a day when they completed the survey, this can impact the outcomes.

Further research is needed to study virtual interventions of this nature. Longer interventions are needed to study the effect of dietary adherence on participants. Completing a power analysis to determine sample size will optimize the significance testing.

## Conclusion

With the virtual shifts we are seeing happen in our society, and the detrimental effects of COVID-19 on mental health (7), this study helps to provide the framework for an effective intervention strategy that can be implemented in worksites, and potentially other settings, to help improve diet quality and improve overall mental wellness including depressive symptoms, anxiety, sleep, social relationships, satisfaction with life, and stress. In addition, worksite interventions have been shown to improve employee productivity and improve lifestyle behavior changes (20, 21). With the positive results from this intervention, we can infer it would result in productive work even during stressful times such as those experienced during COVID-19. Although we did not see more positive mental health outcomes with increased Blue Zones knowledge, we do infer that the Blue Zones Knowledge played an important role in the outcomes seen. Although further research is needed, we conclude that a weekly Blue Zones-based virtual worksite nutrition intervention including weekly cooking sessions and education paired with weekly MI-based small group counseling can help to improve overall diet quality and mental wellness.

## Data Availability Statement

The raw data supporting the conclusions of this article will be made available by the authors, without undue reservation.

## Ethics Statement

The studies involving human participants were reviewed and approved by Northern Arizona University Institutional Review Board. The patients/participants provided their written informed consent to participate in this study.

## Author Contributions

CH, VS, and JS contributed to the design and implementation of the study. NL performed the statistical analysis. CH and NL wrote the first draft of the manuscript. All authors contributed to the manuscript revision and as well as read and approved the submitted version.

## Funding

This work was supported by Eric M. Lehrman 2015 Trust and the Northern Arizona University Department of Health Sciences.

## Conflict of Interest

The authors declare that the research was conducted in the absence of any commercial or financial relationships that could be construed as a potential conflict of interest.

## Publisher's Note

All claims expressed in this article are solely those of the authors and do not necessarily represent those of their affiliated organizations, or those of the publisher, the editors and the reviewers. Any product that may be evaluated in this article, or claim that may be made by its manufacturer, is not guaranteed or endorsed by the publisher.
